# Non‐volatile and Secure Optical Storage Medium with Multilevel Information Encryption

**DOI:** 10.1002/advs.202408287

**Published:** 2024-10-16

**Authors:** Jie Shao, Xiyang Li, Meng Liu, Haiqin Sun, Dengfeng Peng, Fuchi Liu, Qiwei Zhang

**Affiliations:** ^1^ College of Physics and Technology Guangxi Normal University Guilin 541004 China; ^2^ Key Laboratory of Optoelectronic Devices and Systems of Ministry of Education and Guangdong Province College of Physics and Optoelectronic Engineering Shenzhen University Shenzhen 518060 China

**Keywords:** information security, light‐induced valence, multilevel optical storage, photochromism

## Abstract

Non‐volatile photomemory based on photomodulated luminescent materials offers unique advantages over voltage‐driven memory, including low residual crosstalk and high storage speed. However, conventional materials have thus far been volatile and insecure for data storage because of low trap depth and single‐level storage channels. Therefore, the development of a novel non‐volatile multilevel storage medium for data encryption remains a challenge. Herein, a robust, non‐volatile, multilevel optical storage medium is reported, based on a photomodulated Ba_3_MgSi_2_O_8_:Eu^3+^, which combined the merits of light‐induced valence (Eu^3+^ → Eu^2+^) and photochromic phenomena using optical stimulation effects, accompanied by larger luminescent and color contrasts (>90%). These two unique features provided dual‐level storage channels in a single host, significantly improving the data storage security. Notably, dual‐level optical signals could be written and erased simultaneously by alternating 265 and 365 nm light stimuli. Theoretical calculations indicated that robust color centers induced by intrinsic interstitial Mg and vacancy defects with suitable trap depths enable excellent reversibility and long‐term storage capability. By relying on different luminescent readout mechanisms, the encrypted dual‐level information can be accurately decrypted by separately probing the Eu^2+^ and Eu^3+^ signals, thus ensuring information security. This study proposes a novel approach for constructing multilevel information storage channels for information security.

## Graphical abstract

Based on unique features of the photomodulated Ba_3_MgSi_2_O_8_:Eu medium: photo‐induced valence (PV) and photochromic (PC) phenomena, a robust, non‐volatile, and multilevel photomemory is successfully designed. The dual‐level storage channels are written and erased simultaneously by alternating 265 and 365 nm stimuli. The encrypted information can be decrypted by separately probing the Eu^2+^ and Eu^3+^ signals, thus ensuring information security.

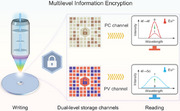

## 1 Introduction

Data storage is a great challenge in the digital information age, and current magnetic storage devices cannot store the massive amounts of information that will be required in the future.^[^ ^1^ ^]^ Optical data storage technology provides an effective solution to these problems because of its low energy consumption, long lifetime, and super‐high capacity.^[^ ^2^ ^]^ Available optical storage techniques include near‐field optical storage, far‐field super‐resolution recording, and polarized light information multiplexing.^[^ ^3^ ^]^ However, these optical storage technologies suffer from the limitations of expensive equipment and complex operations. In recent years, researchers have realized optical storage using photoresponsive luminescent materials such as persistent luminescent materials and photostimulated luminescent materials.^[^ ^4^ ^]^ Because of their abundant electron traps, these materials exhibit excellent photon storage capabilities under short‐wave excitation. Nevertheless, excessively low trap depths render the information stored at room temperature unstable, and volatile information is incompatible with the purpose of optical information storage.

Emerging nanomaterials offer numerous avenues for data loss prevention solutions. The optical properties of graphene oxide can be modulated by changing the structure of the oxygen‐containing functional groups; such changes are highly thermally stable and photo‐unbleachable, ensuring long‐term data storage.^[^ ^5^ ^]^ Zhao et al. developed a photoresist film based on aggregation‐induced emission dyes for stable luminescent information storage using femtosecond laser beams with petabit‐level capacity.^[^ ^6^ ^]^ Xie et al. achieved a multicolor afterglow of NaYF_4_:Ln^3+^ nanoparticles using a surface‐passivated strategy for optical information storage.^[^ ^7^ ^]^ Therefore, maintaining the bistability of matter during light‐matter interactions is an effective strategy for non‐volatile optical storage. However, the harsh production conditions significantly limit the use of nanomaterials as future optical storage materials. Recently, inorganic photochromic (PC) materials have achieved non‐volatile storage and possess unique advantages, including excellent fatigue resistance, thermal stability, and low‐cost characteristics.^[^ ^8^ ^]^ For example, PC glass with a high color‐changing contrast was developed for reversible 3D optical information storage, allowing the stored information to remain undamaged over a long period of time.^[^ ^9^ ^]^ In addition, recently reported light‐induced ionic valence changes have potential applications in X‐ray dose detection, anti‐counterfeiting encryption, and optical information storage.^[^ ^10^ ^]^ Non‐volatile invisible light storage in a dark field can be realized by utilizing the emission intensity difference between lanthanide ions with different valence states. Although some advances have been made in photoresponsive materials for non‐volatile optical storage, they have all been realized by exploiting a single physical property, and thus remain limited in terms of storage capacity and security. In recent years, multiplexing technologies have been proposed that can further increase data storage capacity and information encryption level.^[^ ^11^ ^]^ Multiplexing one or more information levels allows explosive growth in the data capacity, such as angular momentum, optical polarization, and luminescence information (intensity and wavelength).^[^ ^12^ ^]^ However, the introduction of new information channels unavoidably requires more complex and different methods during the writing, reading, and erasing of stored information, which are not the desired results. Therefore, the development of a non‐volatile multilevel storage medium for data encryption in a single host may have great application value and development prospects in the field of optical information security but remains a challenge.

**Figure 1:**
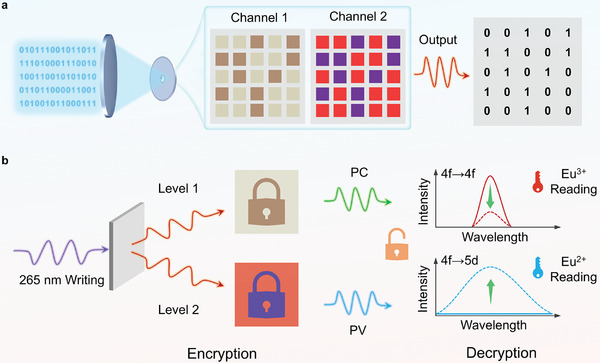
Schematic diagram of multilevel optical information security storage. a) Dual‐channel optical information storage; the brown square (channel 1, PC) is defined as “1”, the blue square (channel 2, PV) is defined as “1”, other spot is defined as “0”. b) Encryption and decryption processes of the dual‐level information storage.

In this study, a novel photomodulated luminescent material was designed by incorporating easily changed‐valence Eu ions into the Ba_3_MgSi_2_O_8_ host. The resulting Ba_3_MgSi_2_O_8_:xEu^3+^ (BMS:xEu) medium exhibited two unique features of photo‐induced valence change (PV) (Eu^3+^ → Eu^2+^) and PC effect, possessing the dual‐channel optical information storage capability with excellent reversibility and non‐volatile storage (**Figure** **1**a). Specifically, it demonstrated that the dual‐channel optical information can be well written and erased by alternating 265 and 365 nm light stimuli, and also quickly read out by measuring the luminescent signals of the Eu^3+^ and Eu^2+^ ions. In particular, multilevel information storage channels significantly improve information storage security (Figure 1b). The underlying mechanisms behind such a multilevel information storage process were elucidated using theoretical calculations and experimental results. We believe that this study will open a new avenue for developing a non‐volatile optical storage medium with multilevel information encryption.

## 2 Results and Discussion

### 2.1 Phase Structural Analysis

**Figure 2:**
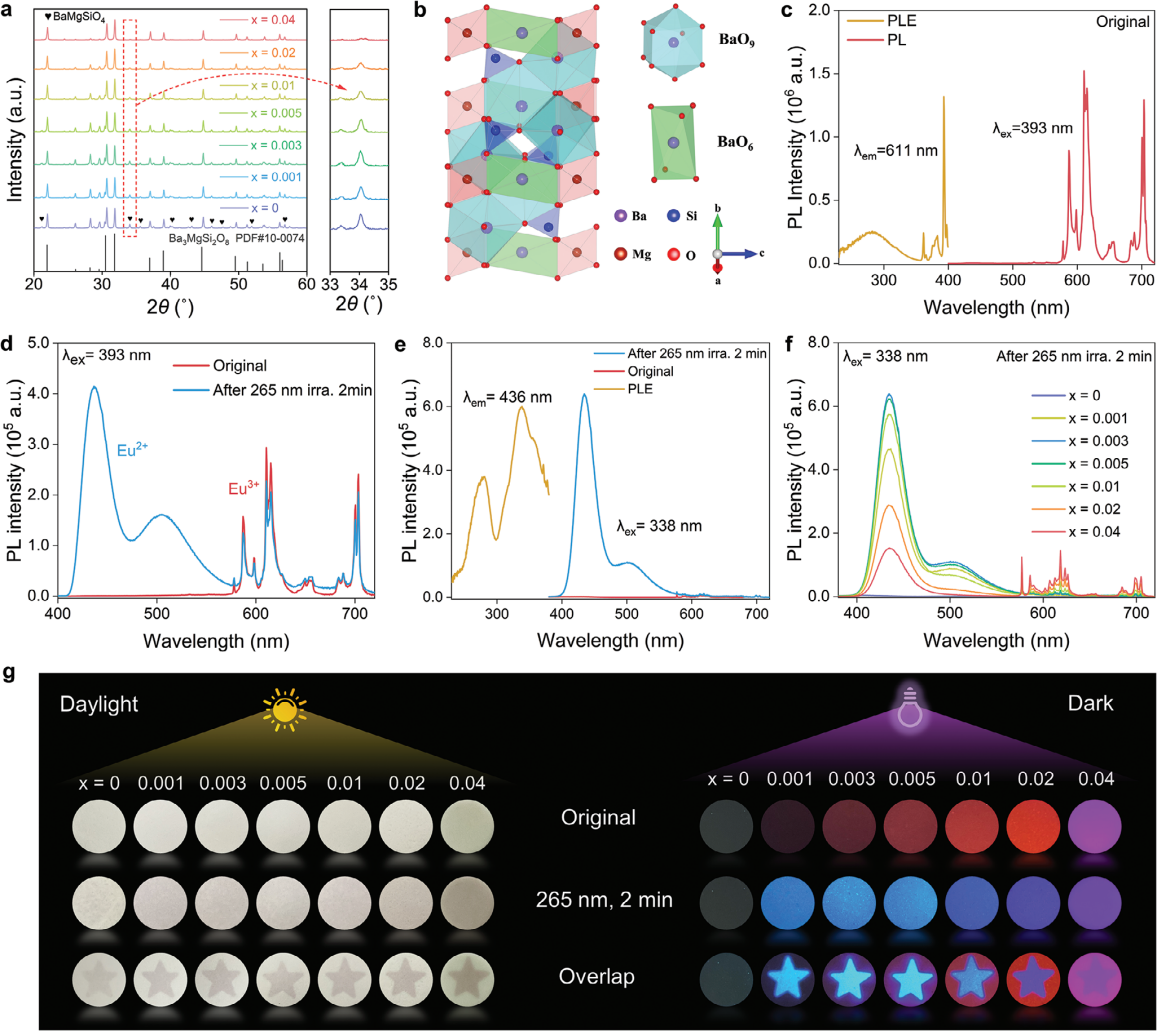
Phase structure analysis and luminescent modulation behaviors of BMS:xEu materials. a) XRD patterns of BMS:xEu samples. b) The crystal structure of Ba_3_MgSi_2_O_8_. c) PLE (λ_em_ = 611 nm) and PL (λ_ex_ = 393 nm) spectra of original BMS:0.003Eu. d) PL spectral changes (λ_ex_ = 393 nm) of BMS:0.003Eu before and after 265 nm irradiation for 2 min. e) PLE (λ_em_ = 436 nm) and PL (λ_ex_ = 338 nm) spectra of BMS:0.003Eu samples after 265 nm irradiation for 2 min. f) PL spectra (λ_ex_ = 338 nm) of BMS:xEu after 265 nm irradiation for 2 min. g) Photographs of the BMS:xEu samples before and after 265 nm irradiation in the bright field (daylight, left) and dark field (365 nm excitation, right).

**Figures** **2**a and S1a (in Supporting Information) show the X‐ray diffraction (XRD) patterns of the Ba_3_MgSi_2_O_8_:xEu^3+^ (BMS:xEu) samples. The main diffraction peaks of all samples can be indexed to the Ba_3_MgSi_2_O_8_ phase (PDF#10‐0074) with a low‐symmetry *P* $\bar{3}$(Z = 3) structure (orthorhombic phase).^[^ ^13^ ^]^ Eu doping did not significantly affect the formation of the main phase structure, indicating that Eu ions were successfully doped into the host lattice. In addition, a minor amount of BaMgSiO_4_ phase (PDF#16‐0573) located at 2θ = 34.1° appeared owing to the instability of Ba_3_MgSi_2_O_8_ during the high‐temperature sintering process (Figure S1b, Supporting Information). The second phase (BaMgSiO_4_) gradually decreased with increasing Eu doping concentration, possibly stemming from the formation of more defects induced by the heterovalent substitution of Eu ions at high concentrations and resulting in a phase transformation from the stable cubic phase (BaMgSiO_4_) to an unstable orthorhombic phase (Ba_3_MgSi_2_O_8_).^[^ ^14^ ^]^ The detailed crystal structure is shown in Figure 2b. The BMS orthorhombic phase is a layered glaserite‐type structure, wherein Ba atoms are present in the form of [BaO_9_] dodecahedra and [BaO_6_] octahedra, and [MgO_6_] octahedra and [SiO_4_] tetrahedra share vertices with each other.^[^ ^14^, ^15^ ^]^ However, the BMS structure was slightly distorted from the ideal $P\bar{3}m1$ (Z = 1) symmetry, further confirming the instability caused by structural distortion.^[^ ^16^ ^]^ This special structure significantly contributed to the photochromism and light‐induced ionic valence.

### 2.2 PV Reaction for Optical Storage

The luminescence modulation behavior of a representative sample (x = 0.003) was determined by the photo‐induced valence (PV) reaction of Eu^3+^. The photoluminescence excitation (PLE) and emission (PL) spectra of the original sample (x = 0.003) are shown in Figure 2c. The PLE spectrum exhibited a weak broadband excitation peak centered at 280 nm and a strong narrowband excitation peak at 393 nm, that typically originate from the electron transfer of O^2−^ → Eu^3+^ ions and the absorption of Eu^3+^ activator, respectively.^[^ ^10b,c^ ^]^ The narrow‐band red emission peaks at 611 and 703 nm were attributed to the typical ^5^D_0_ → ^7^F_2_ and ^5^D_0_ → ^7^F_4_ transitions of Eu^3+^, respectively. As Eu concentration increased above x = 0.04 (Figure S2, Supporting Information), the red emission intensity of Eu^3+^ exhibited a concentration‐dependent quenching effect. The blue emission of Eu^2+^ began to appear with a higher doping concentration of x *≥* 0.04. Generally, a high concentration of heterovalent substitution facilitates the self‐reduction of Eu ions during high‐temperature sintering, and the relevant mechanism can be explained by the theory of charge compensation.^[^ ^17^ ^]^

In particular, the emission spectra of BMS:xEu (0.001 ≤ x ≤ 0.02) after 265 nm light irradiation (47.5 mW · cm^−2^) exhibited a new strong blue emission peak at 436 nm (λ_ex_ = 393 nm), as shown in Figures 2d and S3a–f (Supporting Information). This blue emission is consistent with the PL spectra of the sample sintered in the reduced atmosphere (Figure S4, Supporting Information), suggesting that the emerging blue emission originated from the Eu^2+^ ions formed by the light‐induced reduction of Eu^3+^. As the Eu content was increased above x = 0.04, the light‐induced reduction behavior of Eu^3+^ → Eu^2+^ was completely inhibited. Nevertheless, the red emission intensity located at 611 nm (Eu^3+^) decreased after 265 nm light irradiation, and the PL quenching degree (the PL difference before and after irradiation) gradually increased with the Eu concentration. The PL quenching behavior of Eu^3+^ can be ascribed to a synergistic reaction between the light‐induced Eu^3+^ reduction and photochromism.^[^ ^8b,c^ ^]^

To further investigate the light‐induced reduction behavior, the PLE and PL spectra of BMS:0.003Eu after 265 nm irradiation were characterized (Figure 2e). The PLE spectrum monitored at 436 nm exhibited a new broadband from 220 to 400 nm, corresponding to the 4f^7^ → 4f^6^5d^1^ transition of Eu^2+^.^[^ ^18^ ^]^ The PL spectrum excited at 338 nm contained a main peak at 436 nm and a shoulder peak at 505 nm. Based on the valence and ionic radii of Eu ions, they are most likely to occupy Ba lattice sites at two different positions to form two different emission centers. Considering the crystal‐field splitting effect and ion coordination environment, the short‐wavelength emission located at 436 nm originated from [BaO_6_], and the long‐wavelength emission at 505 nm corresponded to [BaO_9_].^[^ ^19^ ^]^ Such light‐induced reduction behavior only occurred in the samples with x<0.04, as shown in Figure S3f (Supporting Information). As the Eu doping concentration was increased to x = 0.003, the sample exhibited the strongest blue emission (Figure S3g,h, Supporting Information). In contrast, blue emission was not observed for the undoped sample after excitation at 338 or 393 nm (Figure S3a, Supporting Information). Furthermore, these photoresponsive characteristics can be visually displayed by a “star” pattern in bright and dark fields, as shown in Figure 2g. The original samples without light irradiation emitted the red color of Eu^3+^ ions upon 365 nm excitation (dark field). After 265 nm irradiation, the samples displayed a bright blue emission of Eu^2+^. Correspondingly, the surface color of the samples changed from white to brown under daylight, showing typical PC behavior.^[^ ^13^, ^14^ ^]^ The larger color contrasts based on the two unique features of PV and PC reactions make BMS:xEu an excellent candidate for designing high‐level optical storage memory.

**Figure 3:**
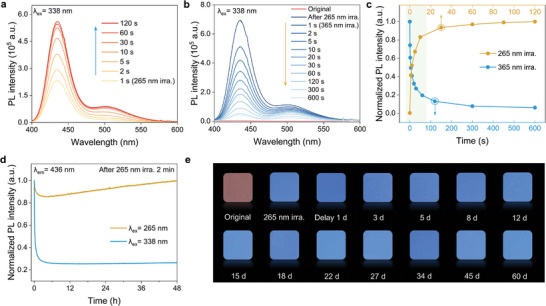
Optical storage behaviors of light‐induced redox reactions for BMS:xEu. a) PL spectral variations (λ_ex_ = 338 nm) of BMS:0.003Eu by 265 nm “writing” (47.5 mW · cm^−2^) at different time intervals. b) PL spectral variations (λ_ex_ = 338 nm) of the irradiated sample by 365 nm “erasing” (218.9 mW · cm^−2^) at different time intervals. c) Dynamic photoresponse processes of the normalized emission intensity located at 436 nm by 265 and 365 nm stimuli at different time intervals. d) Time‐dependent curves of PL intensity (436 nm) by in situ continuous excitation of 265 nm (27.0 mW · cm^−2^) and 338 nm (190.0 mW · cm^−2^). e) Luminescent images (λ_ex_ = 365 nm) of BMS: 0.01Eu after 265 nm irradiation for 2 min with different delaying days.

To estimate the potential advantages of BMS:xEu in optical storage applications, the dynamic reversible processes of the photoresponsive behavior were investigated. As shown in **Figures** **3**a and S5 (Supporting Information), the blue emission intensity of BMS:0.003Eu at 265 nm gradually increased with irradiation time, indicating that more Eu^3+^ ions were reduced to Eu^2+^. Nevertheless, it was saturated when the irradiation time reached 120 s. In addition, the reduced Eu^2+^ ions could return to their original state (Eu^3+^) upon 365 nm illumination (218.9 mW · cm^−2^) or thermal stimulus because the photogenerated Eu^2+^ ions were often present in a metastable state,^[^ ^16^ ^]^ and the corresponding blue emission quenched or disappeared. For example, the recovery process of Eu^2+^ ions upon 365 nm illumination and thermal treatment is presented in Figures 3b and S6 (Supporting Information), demonstrating that thermal treatment is more effective and can realize a total recovery, while a small and weak emission band remained after 365 nm illumination for 600 s. However, complete recovery requires more energy via a higher heat treatment temperature at 300 and 500 °C (in Figure S6, Supporting Information), which also implies the excellent temperature stability of the photogenerated Eu^2+^ ions. In Figure 3c, the normalized blue emission intensity (436 nm) at different irradiation times clearly shows the dynamic reversibility between the Eu^2+^ and Eu^3+^ ions. In the fast‐response stage, the PL intensity change reached 80%. This highly sensitive photoresponse is convenient for optical storage.

From the perspective of practical applications, the cycling reliability of the photoinduced valence behavior in BMS:xEu is a crucial factor. Figure S7 (Supporting Information) presents the blue emission intensity of BMS:0.003Eu as a function of the cycle number upon alternating 265 and 365 nm illumination or thermal recovery. The blue emission at 436 nm showed no evident degradation after 10 cycles, indicating excellent repeatability. Additionally, the written information by 265 nm irradiation could be effectively read out by tracing Eu^2+^ emission (λ_ex_ = 265 nm) without any interference (Figure 3d). Although the photogenerated metastable Eu^2+^ ions easily transform into Eu^3+^ upon in situ continuous light stimuli (λ_ex_ = 265 nm) in its initial time interval for the 265 nm irradiated sample, the oxidized Eu^3+^ could restore its original stage once with further extension of the luminescent readout time. Nevertheless, continuous information readout is unfavorable for the excitation wavelength of 338 nm, as it will bleach Eu^2+^ → Eu^3+^. Therefore, it is essential to realize the effective readout of the “writing” optical information by choosing appropriate excitation wavelengths of activators. To verify the stability of the photogenerated Eu^2+^ ions at room temperature, the luminescent images (λ_ex_ = 365 nm) of Eu^2+^ were displayed with different delaying times, as shown Figure 3e. The sample without light irradiation showed a reddish‐brown color of Eu^3+^. After 265 nm irradiation for 2 min, a strong blue color was clearly observed by the naked eye, which remained almost unchanged after two months (60 d). These results indicate that the photogenerated Eu^2+^ ions in the BMS matrix possess excellent luminescent stability and provide an effective information channel for realizing non‐volatile optical storage.

### 2.3 PC Reaction for Optical Storage

**Figure 4:**
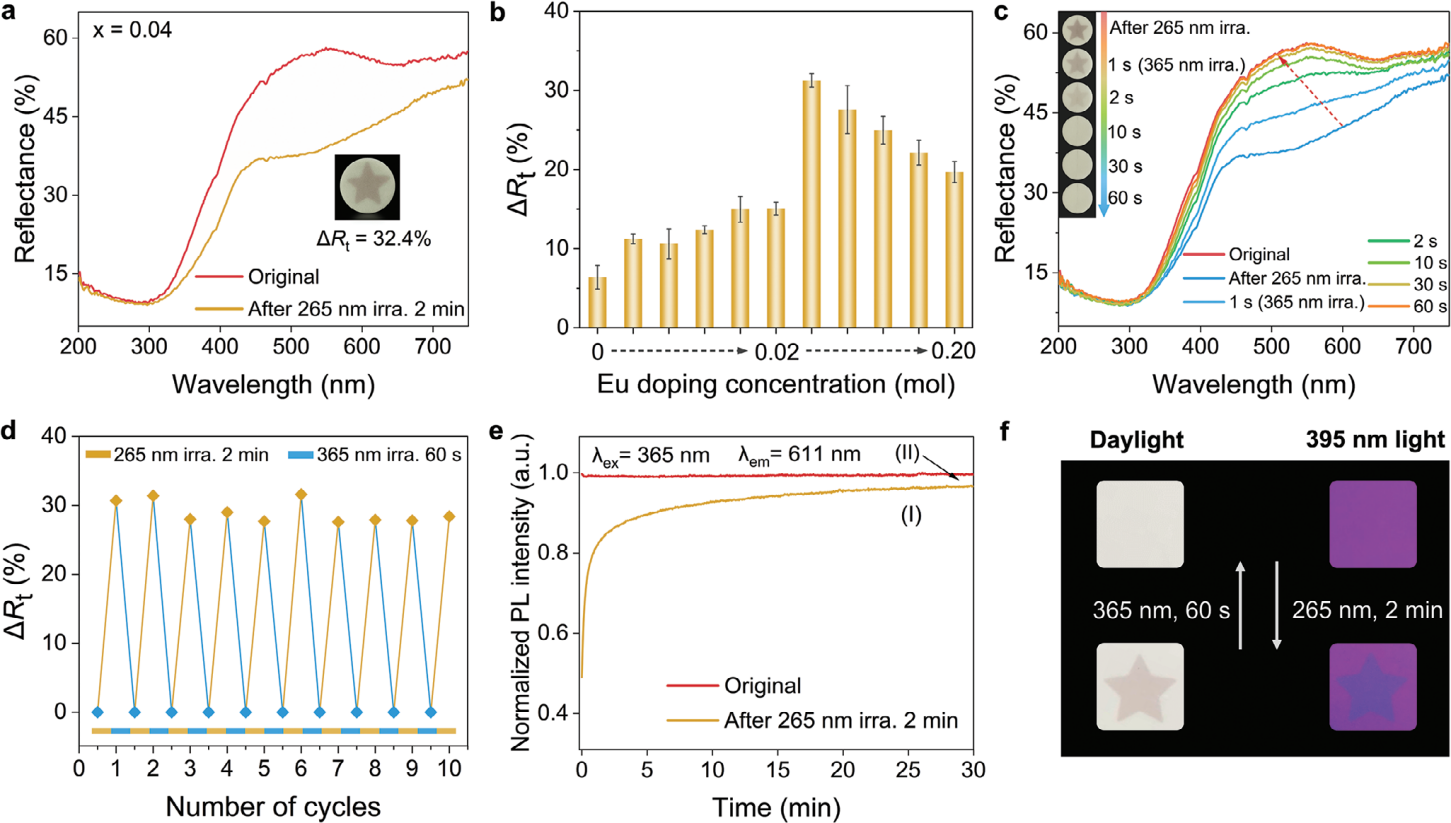
Optical storage behaviors of PC reactions for BMS:xEu. a) Reflectance spectra of the representative sample (x = 0.04) before and after 265 nm irradiation for 2 min, the inset is the PC image of a “star” pattern (the irradiated area). b) The Δ*R*_t_ variations with Eu doping concentration located at 515 nm. c) Reflectance spectra of the colored sample under 365 nm irradiation at different time intervals, the inset is the bleaching PC images. d) 10 reversible cycles of Δ*R*_t_ values by alternating 265 and 365 nm irradiation. e) Normalized PL intensity variation of Eu^3+^ (611 nm) with excitation time under 365 nm in situ continuous excitation (102.3 mW · cm^−2^) for the original (red line) and colored sample (yellow line) (x = 0.04). f) PC and luminescent modulation images of BMS:xEu (x = 0.02) after 265 nm irradiation for 2 min (left) and 365 nm irradiation for 60 s (right).

Photochromic (PC) behavior always involves a series of changes in the surface color, diffuse reflectance, and luminescence properties. As shown in **Figures** **4**a and S8 (Supporting Information), all samples showed a significant decrease in reflectivity at 400–750 nm after irradiation at 265 nm for 2 min. The “star” pattern can be seen clearly in daylight (inset of Figure 4a). To better quantify the degree of photochromism, the PC efficiency (∆*R*_t_) can be defined with Equation (1):

(1) $$\Delta R_{t}=\left(R_{0}-R_{1}\right)/R_{0}\times 100\%$$

where *R*_0_ and *R*_1_ are the reflectance intensities of BMS:xEu before and after 265 nm irradiation for 2 min, respectively. Compared with the undoped sample, an enhancement of the photochromism efficiency (∆*R*_t_) was achieved in all Eu‐doped samples (Figure 4b; Table S1, Supporting Information). The sample with x = 0.04 exhibited the optimized PC efficiency (∆*R*_t (mean)_ = 31.26% at 515 nm,). The abrupt change of the Δ*R*_t_ value (x = 0.04) may have resulted from the formation of Eu^3+^ sublattice and the suppression of light‐induced Eu^3+^ to Eu^2+^ at high doping concentration that promoted electron transfer to the photochromic units through defect‐assisted energy migration.^[^ ^16^, ^20^ ^]^ Figure 4c shows the fast bleaching or erasing process of the colored sample (x = 0.04) under 365 nm light irradiation. The reversibility of reflectivity was determined for all samples. The decreased reflectivity rapidly increased until it returned to its initial stage, and the corresponding “star” PC pattern was hardly visible to the naked eye upon 365 nm irradiation for 10 s. After 365 nm light irradiation for 60 s, the decreased reflectance completely recovered to its original state, as observed in the PC images (inset of Figure 4c). The photoresponse bleaching time of PC was far faster than that of the Eu valence change (Eu^2+^ → Eu^3+^). Such a sensitivity difference provides great possibilities for the encryption and decryption of optical data storage. In addition, the PC reaction was reversible. As shown in Figure 4d, the ∆*R*_t_ value was completely reversible via 265 nm irradiation for 2 min and 365 nm irradiation for 60 s. After 10 reversible cycles, the ∆*R*_t_ value showed only a slight change, demonstrating the material's optical storage capability with excellent repeatability.

The “writing” and “erasing” processes based on the PC reaction could be read out by detecting Eu^3+^ PL changes. Figure 4e depicts the dynamic variations in the red emission intensity (611 nm) with the excitation time under in situ continuous excitation at 365 nm for the original or colored sample (x = 0.04). For the original sample without 265 nm irradiation, the PL intensity at 611 nm did not change as the excitation time increased, implying that the 365 nm wavelength did not participate in the PC reaction. Nevertheless, the red emission significantly decreased after 265 nm irradiation for 2 min, and the decreased PL intensity reached 50.9% of the original state. The decreased red emission intensity returned to its original level when the 365 nm excitation time was increased, owing to its bleaching effect. During luminescence modulation, the PL intensity changes in region (I) are mainly attributed to luminescence recovery induced by the PC reaction, whereas only a few contributions arise from the reduction of Eu^3+^ to Eu^2+^, as shown in the PL intensity gap of region (II). In contrast, the PC reaction did not influence the blue emission intensity of Eu^2+^ (Figure S3f, Supporting Information). These results suggest that dual‐level information stored through light‐induced valence and photochromism can be accurately distinguished and separately read out by detecting the luminescent signals of Eu^2+^/Eu^3+^ ions. Similarly, the PC and luminescent modulation behaviors of the samples could be visualized in the related photographs. Figure 4f shows the luminescent photochromism photographs of BMS:0.02Eu in dark and bright fields during the writing (265 nm), erasing (365 nm), and reading (395 nm) processes. Except for the typical PC phenomenon in the bright field, the larger luminescence contrast of Eu^3+^ in the dark field (“star” pattern) is clearly observed. Importantly, this photochromism‐induced red emission modulation provides a new channel for non‐volatile optical information storage.

### 2.4 Optical Storage Mechanisms

**Figure 5:**
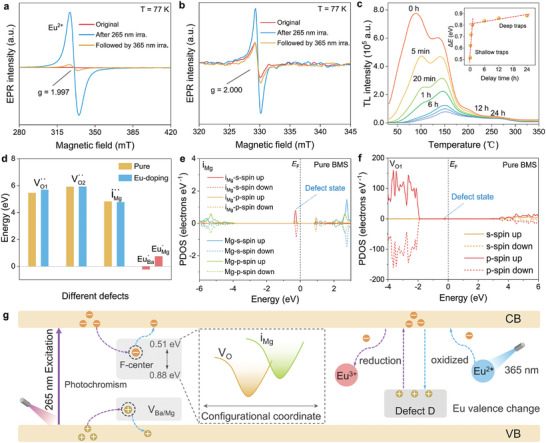
Optical storage mechanisms of the BMS:xEu system. a) EPR spectra of Eu^2+^ ions in BMS:0.003Eu under different irradiation conditions. b) EPR spectra of oxygen vacancy in BMS:0.003Eu under different irradiation conditions. c) TL spectra of BMS:0.003Eu after mercury lamp irradiation at different delay time intervals. The inset shows a plot of trap depth versus delay time. d) Formation energies of different defects including oxygen vacancy, interstitial Mg, and Eu substitution sites (Ba and Mg). e) and f) The calculated PDOS of interstitial Mg, and oxygen vacancy defects, respectively. g) Schematic diagram of light‐induced electron transfer and defect‐assisted charge carrier capture in photochromism and light‐induced valence behaviors.

The underlying mechanisms of optical storage in BMS:xEu were further elucidated using electron paramagnetic resonance (EPR) spectroscopy, thermoluminescence (TL) spectroscopy, and theoretical calculations. **Figure** **5**a shows in situ EPR spectra of Eu^2+^ ions under different light irradiation conditions. Notably, no EPR signal for Eu^2+^ was observed in the original sample, whereas a strong EPR signal was detected after 265 nm irradiation, implying that the blue emission originated from the photogenerated Eu^2+^ ions (Figure 2d).^[^ ^10c^ ^]^ Following irradiation at 365 nm, the EPR signal decreased significantly, indicating the transformation from Eu^2+^ to Eu^3+^. However, trace amounts of Eu^2+^ were not fully recovered, consistent with the PL measurements (Figure 3b). Such a redox process inevitably involves charge carrier transfer with a defect‐assisted effect, and the formed defects in BMS:xEu played an important role in determining the optical storage.^[^ ^4^, ^10^ ^]^ As shown in Figure 5b, the EPR signal of oxygen vacancy ($V_{o}^{\cdot \cdot }$) was present in the spectrum of the original sample. After 265 nm irradiation, the EPR signal of $V_{o}^{\cdot \cdot }$ increased in intensity, indicating that the light irradiation induced more oxygen vacancies that resulted in the formation of PC F‐centers.^[^ ^14^ ^]^ Upon 365 nm bleaching, the EPR signal of $V_{o}^{\cdot \cdot }$ completely recovered to its original level. Therefore, the oxygen vacancy defects in BMS:xEu may contribute to the valence change or photochromism by the defect‐assisted photoinduced electron transfer process.^[^ ^21^ ^]^

To reveal the trap depth distribution, TL spectra were measured over a broad temperature range from 30 to 350 °C with different delay times (Figure 5c). With increasing delay time, the TL intensity gradually decreased and then became saturated within 24 h. Based on the TL results, we fitted the TL curves with different delay times using an initial rise analysis method (Equation 2).^[^ ^22^ ^]^ The fitted curves are shown in Figure S9 (Supporting Information):

(2) $$I\left(T\right)=Cexp\left(\frac{\Delta E}{kT}\right)$$

where *I* is the TL intensity, *C* is the fitting constant, *k* is the Boltzmann constant, *T* is the temperature in *K*, and ∆*E* is the trap depth. As shown in the inset of Figure 5c, the obtained trap depth distribution covered a wide range of 0.51–0.88 eV, and exhibited two different slopes: the shallow traps for fast carrier release (0–1 h) and the deep traps for slow carrier release (1–24 h). This result is consistent with the photoresponse sensitivity to 365 nm irradiation during the PC bleaching and photo‐induced Eu^2+^ → Eu^3+^ oxidation processes, suggesting that the shallow and deep traps correlate with electronic capture defects and contribute to the photochromism and photo‐induced valence change.

To further understand the origin of the defect‐induced traps, the relevant defect formation energies were calculated using density functional theory, as shown in Figure 5d. According to the calculated formation energies of $\;Eu_{Ba}^{\cdot }$ (−0.23 eV) and $Eu_{\mathrm{Mg}}^{\cdot }$ (0.75 eV), Eu atoms preferentially occupy the Ba position, which is fully consistent with the designed compositions. For pure or Eu‐doped samples, the formation energy of interstitial Mg defects ($i_{Mg}^{\cdot \cdot }$) is always lower than that of the $V_{o}^{\cdot \cdot }$ defects (*V*_O1_ and *V*_O2_). This means that more. $i_{Mg}^{\cdot \cdot }$ defects would be generated during high‐temperature sintering, accompanied simultaneously by the creation of corresponding $V_{Mg}^{\prime \prime }$ defects acting as electron/hole traps in the matrix. Especially, the formed energy levels of defect states from electron traps were determined to be located between the valence band (VB) and conduction band (CB) by calculating the partial density of states (PDOS) of $i_{Mg}^{\cdot \cdot }$ and $V_{o}^{\cdot \cdot }$ for pure (in Figure 5e,f) and Eu‐doped samples (Figure S10, Supporting Information). The position of the $i_{Mg}^{\cdot \cdot }$ energy level is closer to the CB compared to the $V_{o}^{\cdot \cdot }$ defect state. Combined with the TL analysis (Figure 5c), we concluded that the shallow traps mainly arise from $i_{Mg}^{\cdot \cdot }$ defects and the deep traps correspond to the $V_{o}^{\cdot \cdot }$ defects.

The photoinduced redox process is therefore closely related to the electron transfer between the Eu ions and defects (Figure 5g). When Eu^3+^ ions entered the lattice of Ba_3_MgSi_2_O_8_, every two Eu^3+^ ions replaced three Ba^2+^ ions to maintain electroneutrality.^[^ ^17c^ ^]^ In principle, one vacancy defect of Ba ions and two positively charged defects of ${\mathrm{Eu}}_{\mathrm{Ba}}^{\cdot }$ were formed simultaneously, as shown in Equation (3).

(3) $$2Eu^{3+}+3Ba^{2+}\;\overset{\mathrm{High}\;\mathrm{temperature}}{\to }\;V_{\mathrm{Ba}}^{\prime \prime }{+2\mathrm{Eu}}_{\mathrm{Ba}}^{\cdot }$$

(4) $$Eu^{3+}+D^{n}\overset{265\;nm}{\to }Eu^{2+}+D^{n+1}$$

(5) $$Eu^{2+}+D^{n+1}\overset{365\;nm}{\to }Eu^{3+}+D^{n}$$

The photo‐induced redox process is described by Equations (4) and (5), where n is the charge state of defect D. Stimulated by 265 nm light, Eu^3+^ ions would act as an electron trap to capture electrons from the hole‐capturing defect D^n^, then Eu^3+^ is reduced to Eu^2+^ while a new defect D^n+1^ forms (Equation (4)).^[^ ^10^, ^23^ ^]^ Defect D most likely originated from cationic vacancy‐related defects (such as $V_{Mg}^{\prime \prime }$ and $V_{Ba}^{\prime \prime }$). Additionally, electrons trapped by Eu ions could be released by 365 nm irradiation or annealing. Under 365 nm excitation, the electrons of photoreduced Eu^2+^ in the metastable state were excited from the 4f state to the 5d state and subsequently transferred to the defective D^n+1^, leading to the oxidation of Eu^2+^ to Eu^3+^ (Equation (5))

In addition, the electron‐capturing defects (Such as $i_{Mg}^{\cdot \cdot }$ and $V_{o}^{\cdot \cdot }$) would contribute to the PC reaction. Under 265 nm light irradiation, electrons were excited from the VB to the CB, where they were subsequently captured by electron defect traps, forming color centers.^[^ ^8^, ^20^ ^]^ In contrast, cationic vacancies tend to trap holes in the VB. The formed F‐centers caused strong absorption in the visible region of 400–750 nm, leading to a change in the surface color. Finally, the captured carriers escaped from the F‐centers and returned to their initial state after 365 nm light irradiation. According to energy transfer (ET) theory,^[^ ^21a^ ^]^ luminescence quenching can be ascribed to the ET from luminescent centers (Eu^3+^) to F‐centers, owing to the perfect overlap between the emission band (Eu^3+^) and the absorption band (Figure S11, Supporting Information). The electron energy with excited states from Eu^3+^ is transferred to the F‐centers, resulting in the luminescent quenching of Eu^3+^ by radiative or non‐radiative energy transfer.^[^ ^24^ ^]^

### 2.5 Multilevel Information Encryption Applications

**Figure 6:**
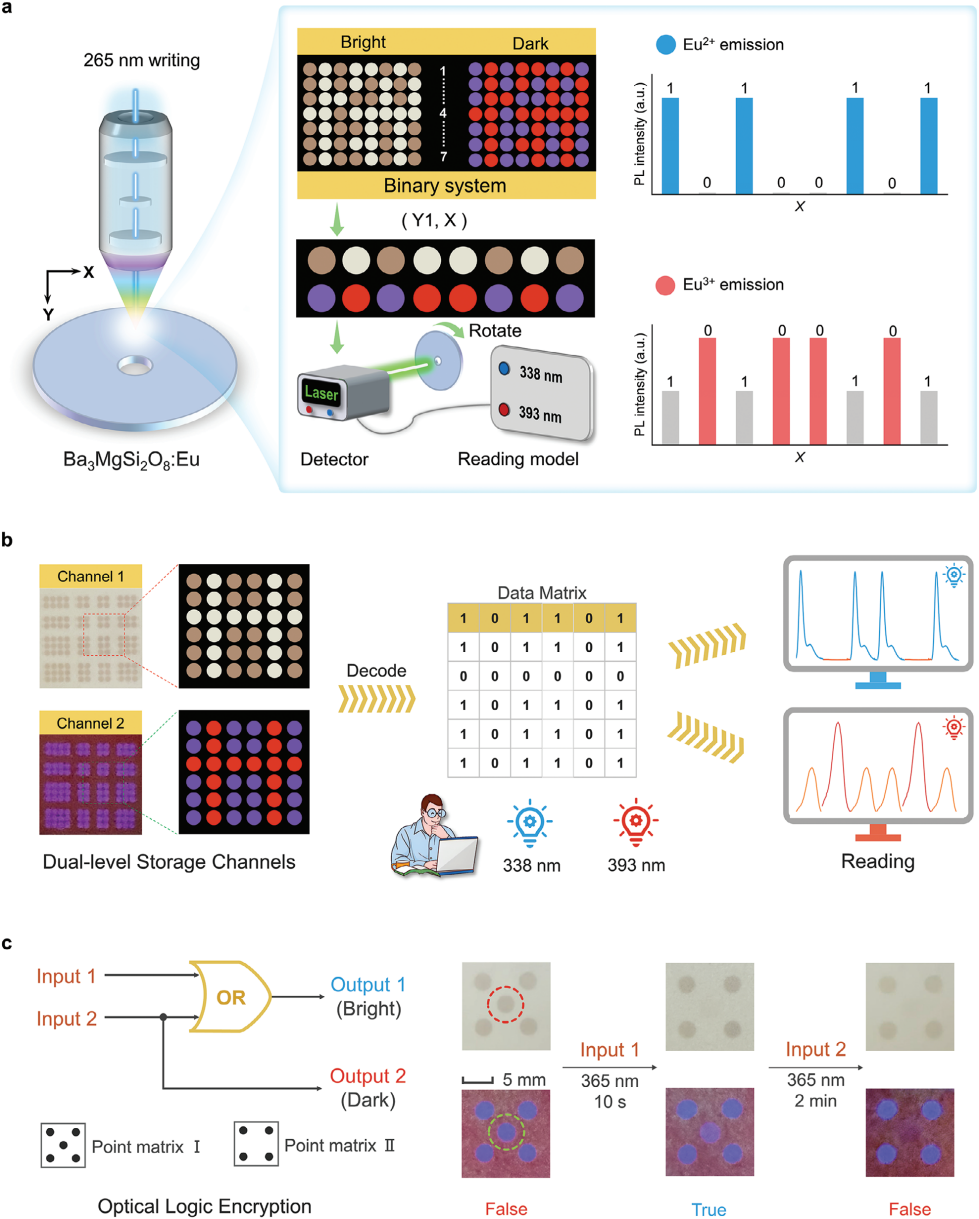
Multilevel information encryption photomemory in BMS:xEu. a) Schematic diagram of the “writing” and “reading” processes for dual‐channel photomemory based on BMS:xEu. b) PC and luminescent photographs of BMS:0.02Eu after the 265 nm bit‐to‐bit scanning along the spot arrays shown in (a). Channel 1 and 2 are PC and PL images in bright field (daylight) and dark field (λ_ex_ = 365 nm), respectively. The superimposed binary information was read out by measuring the Eu^2+^ (λ_ex_ = 338 nm) and Eu^3+^ (λ_ex_ = 393 nm) PL signals. c) Design of optical logic encryption system. The irradiated spot by 265 nm is defined as “1”, and the bleaching spot by 365 nm is defined as “0”. Scale bar: 5 mm.

As a novel optical storage medium, Ba_3_MgSi_2_O_8_:xEu^3+^ exhibited dual‐level optical storage channels of light‐induced valence change and photochromism, and its photoresponse states were highly stable, offering the prospect of nonvolatile multilevel information encryption. In this study, we designed a secure photomemory system with multilevel information encryption and demonstrated a bit‐by‐bit optical storage process. As shown in **Figure** **6**a, the encoded information was written onto a ceramic plate made of BMS:0.02Eu using a confocal laser of 265 nm. The binary system formed by the microregions on the plate contained different information channels in the dark and bright fields. The information array (Y1, X) was read by rotating the information plate and detecting the Eu^2+^/Eu^3+^ PL signals. In general, the information written by 265 nm is defined as “1” and the original information as “0.” When the stored information based on the photoinduced reduction channel was read out by detecting the Eu^2+^ signals, the information from the arrays (Y1, X) occurred at the peak intensity of the Eu^2+^ emission, and vice versa. In addition, the PC channel exhibited a similar signal output when Eu^3+^ signals were detected.

As a proof of concept, the “writing” and “reading” processes of the dual‐level optical information are visually displayed on the ceramic plate of BMS:0.02Eu. Figure 6b shows two photographs captured from the stored dual‐channel information written by the 265 nm laser. One photograph in a bright field represents the information channel of photochromism, which contains the initial white spots (“0”) and the irradiated brown spots (“1”) in different locations. Another photograph of the photoinduced valence channel could only be displayed after 365 nm excitation in the dark field. Therefore, the two types of information were superimposed on the same region of the ceramic plate, then realizing the information encryption. After recording, the microregion information consisted of a “6 × 6” binary array, wherein every written data point in one of the arrays “101101” contained two different information characteristics. Accordingly, the recorded dual‐level information was correctly distinguished and retrieved by separately measuring the Eu^2+^ (λ_ex_ = 338 nm) and Eu^3+^ (λ_ex_ = 393 nm) PL signals.

To further improve the security of the recorded information, we present a practical application of BMS:xEu in optical logic encryption, based on the different photoresponse sensitivities of photochromism and photo‐induced valence change during the “erasing” process of information. An “or” gate‐related logic encryption scheme was designed (Figure 6c). Input 1 and input 2 represent the recorded information with the “365 nm irradiation, 10 s” and “365 nm irradiation, 2 min,” while the output 1 (bright) and output 2 (dark) signals correspond to point matrix I and II, respectively. The resulting optical‐logic encryption truth table is presented in Table S2 (Supporting Information). First, the true information is hidden in point matrix I. The signal input process was used to illuminate the intermediate marker points with 365 nm light for different time intervals (Figure S12, Supporting Information). When the output was point matrix I in a dark field, the match may be point matrix II or point matrix I in a bright field. In addition, when point matrix II was observed in the bright field, it may be point matrix I or point matrix II in the dark field. According to the encryption logic, the true information can only be read when input 1 is “1” and input 2 is “0,” otherwise false information would be displayed. Therefore, the only method to decrypt such an encryption system is to perform the correct input and ensure the security of the optical information.

## 3 Conclusion

In summary, a novel optical storage medium, Ba_3_MgSi_2_O_8_:xEu^3+^, was developed and exhibited the dual‐level optical storage characteristics of PV and PC behaviors. When stimulated with 265 nm light, the samples displayed a distinct valence transition from Eu^3+^ to Eu^2+^, accompanied by a larger luminescent color contrast. Meanwhile, the surface color became brown, and the luminescence of Eu^3+^ decreased because of the PC reaction. In particular, the photoresponse states alternating between 265 and 365 nm were highly stable, providing a good opportunity for nonvolatile multilevel information encryption. The multilevel encoded information could be correctly distinguished and retrieved by detecting the Eu^2+^ and Eu^3+^ luminescent signals. The results of experimental and theoretical calculations demonstrated that interstitial Mg and oxygen vacancy defects play important roles in determining the photoresponsive characteristics, thus facilitating light‐induced electron transfer and charge carrier capture. These unique features are advantageous for the application of the BMS:xEu system in the fields of information security and non‐volatile storage, and this work promotes the development of optical information storage technology.

## Conflict of Interest

The authors declare no conflict of interest.

## Supporting information



Supporting Information

## Data Availability

The data that support the findings of this study are available in the supplementary material of this article.
