# Supplementary material for: Non‐volatile and Secure Optical Storage Medium with Multilevel Information Encryption
Source: Adv Sci (Weinh). 2024 Oct 16;11(45):2408287. doi: 10.1002/advs.202408287 (PMC11615769; doi:10.1002/advs.202408287)
Supplement: Supplementary file 1 — Supporting Information [file ADVS-11-2408287-s001.docx]

Supporting Information

Non-volatile and Secure Optical Storage Medium with Multilevel Information Encryption

Jie Shao, Xiyang Li, Meng Liu, Haiqin Sun^*^, Dengfeng Peng, Fuchi Liu, Qiwei Zhang^*^

**Supplementary Figures and Tables’ List**

1. Experimental Methods and Characterization

B. Supplementary Figures (Figure S1-12)

C. Supplementary Tables (Table S1-2)

1. **Experimental Methods and Characterization**

**Sample fabrication:** A series of Ba_3-_*_x_*MgSi_2_O_8_:*x*Eu (*x* = 0, 0.001, 0.003, 0.005, 0.01, 0.02, 0.04, 0.08, 0.12, 0.16 and 0.20) samples were prepared by high-temperature solid-phase method. The starting materials were Ba_2_CO_3_ (Aladdin, 99.99%), MgO (Aladdin, 99.99%), SiO_2_ (Alfa Aesar, 99.5%), and Eu_2_O_3_ (Aladdin, 99.99%). The raw materials were weighed according to the stoichiometric ratio, mixed and ground 3 times in a mortar with anhydrous ethanol, and then pre-sintered at 1100°C for 4h in air. The pre-sintered powder was again placed in a mortar, mixed with anhydrous ethanol and ground 2 times. 10 wt% polyvinyl alcohol (PVA) is added for granulation. The powders were pressed into a disk of 12 mm in diameter and 1 mm in thickness, and then heated in air at 550 ℃ for 6 h to remove PVA. Finally, the fresh samples were sintered in air at 1350~1400°C for 2 h.

**Structural and optical characterizations:** The phase structure of the samples was characterized using a powder X-ray diffractometer (XRD; MiniFlex600, Rigaku, Japan). The diffuse reflectance spectra were characterized by a UV/Vis spectrophotometer (UV-2700, SHIMADZU, Japan). Photoluminescence excitation (PLE) and emission (PL) spectra were measured using a transient steady-state fluorescence spectrometer (FLS1000, Edinburgh Instruments, UK). Electron paramagnetic resonance (EPR) spectra of Eu^2+^ ions and oxygen vacancies were obtained by an electron paramagnetic resonance spectrometer (EPR; A300, Bruker, Germany). Thermoluminescence (TL) spectra were detected using an SL18 TL analyzer after 3 min of irradiation with a Hg lamp irradiation. The samples were irradiated using commercial diode lasers of 265 nm (47.5 mW·cm^-2^) and 365 nm (218.9 mW·cm^-2^). Other wavelength light signals used to in-situ continuous excitation of 265 nm and 338 nm (Figure 3d), and 365 nm (Figure 4e) were obtained by FLS1000 fluorescence spectrometer, the power densities of them are about 102.3 mW•cm^-2^ (365 nm), 190.0 mW•cm^-2^ (338 nm) and 27.0 mW•cm^-2^ (265 nm), respectively.

**DFT methodology:** All the density functional theory (DFT) calculations are implemented by the VASP code. The GGA-PBE function is chosen as the correlation and exchange potential. The DFT-D3 function is used to analyze the weak van der Waals interaction. The selective cut off energy of the plane-wave is 400 eV. Gamma points in the Brillouin-zone are chosen for integration. Total energies of the systems converge to 10^-5^ eV in the iteration solution of Kohn-Sham equation. The force on each atom reduces to 0.05 eV/Å after geometry optimization. The lattice constant of Ba_3_MgSi_2_O_8_ is calculated to be a=b=9.699 Å and c=7.266 Å. The supercell consists of 2×2×2 unit cells with 336 atoms. Eu substitutions at Mg and Ba sites are considered in Ba_3-_*_x_*MgSi_2_O_8_ crystal, and the oxygen vacancy and interstitial Mg as intrinsic defects.

**B. Supplementary Figures (Figure S1-12)**


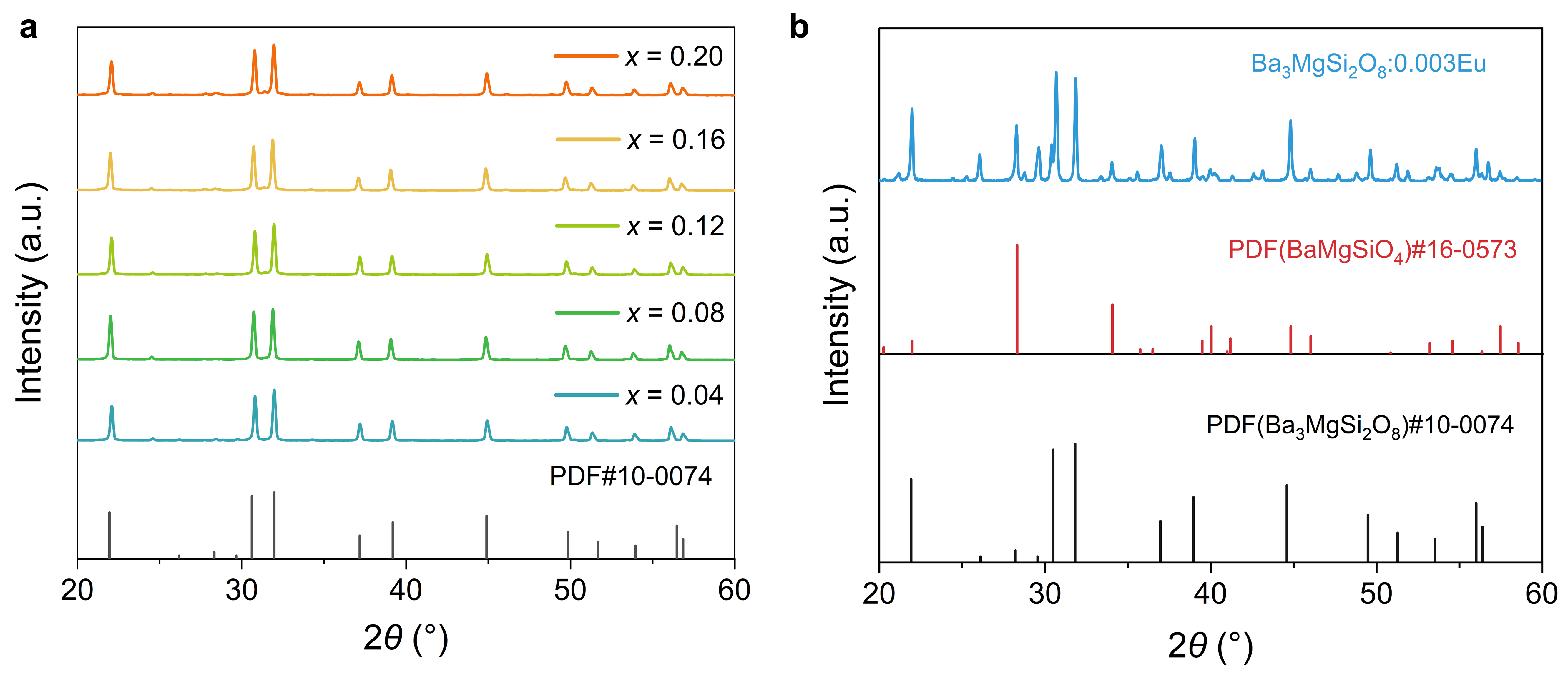


**Figure S1.** (a) XRD patterns of BMS:xEu (*x* = 0.04, 0.08, 0.12, 0.16 and 0.20). (b) XRD patterns and standard PDF cards of pure Ba_3_MgSi_2_O_8_ and BaMgSiO_4_.


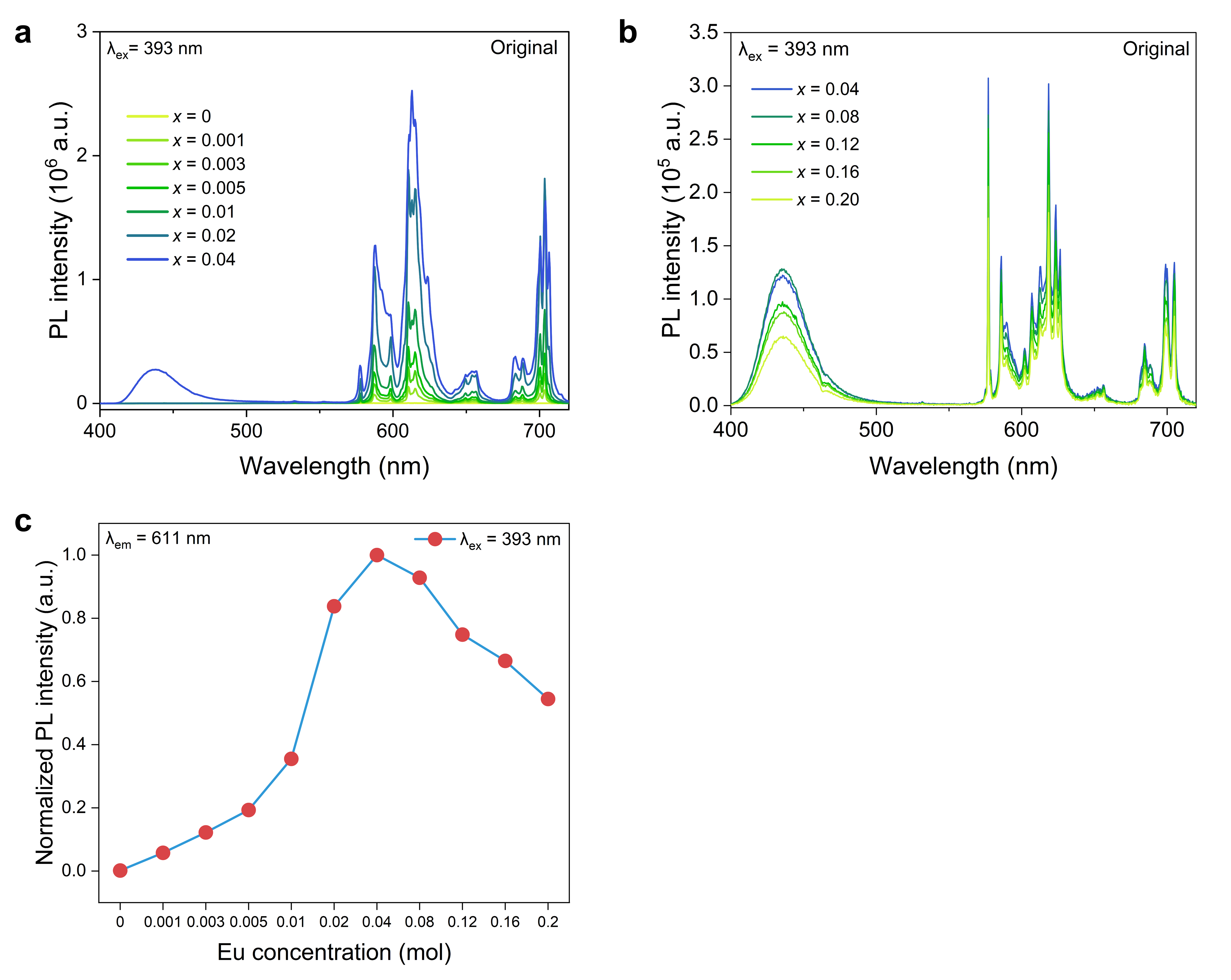


**Figure S2.** (a) and (b) PL spectra (λ_ex_ = 393 nm) of BMS:xEu (x = 0, 0.001, 0.003, 0.005, 0.01, 0.02, 0.04, 0.08, 0.12, 0.16 and 0.20) without any light irradiation. (c) Red emission intensity (611 nm) versus Eu concentration curves of BMS:xEu.


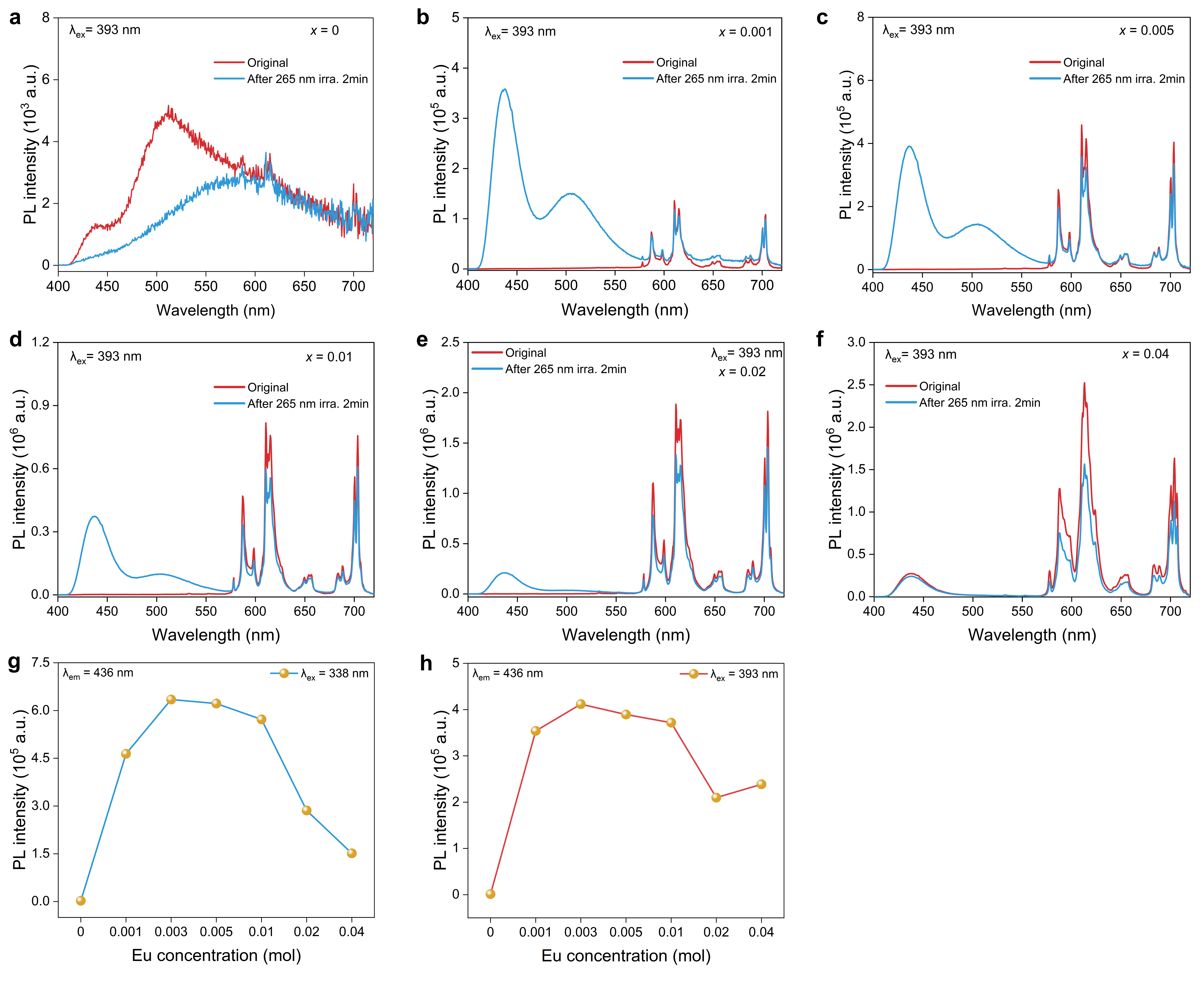


**Figure S3.** (a)-(f) PL spectral changes (λ_ex_ = 393 nm) of BMS:xEu (x = 0, 0.001, 0.005, 0.01, 0.02, and 0.04) before and after 265 nm irradiation 2 min. (g) and (h) Blue emission intensity (436 nm) versus Eu concentration curves of BMS:xEu after 265 nm irradiation 2 min under different excitation wavelengths of 338 nm and 393 nm, respectively.


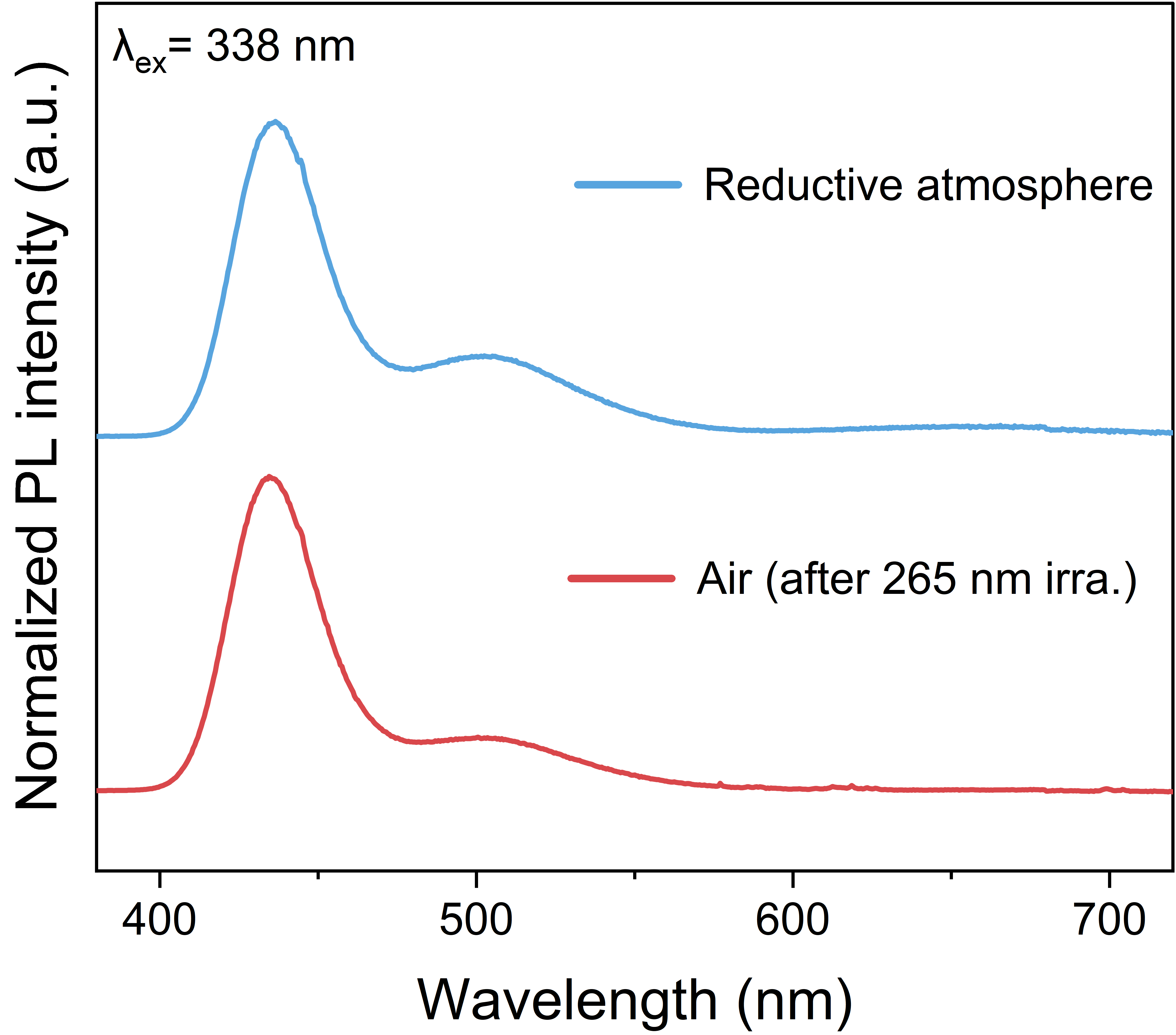


**Figure S4.** Normalized PL spectra (λ_ex_ = 338 nm) of BMS:0.003Eu sintered in air and reducing atmosphere.


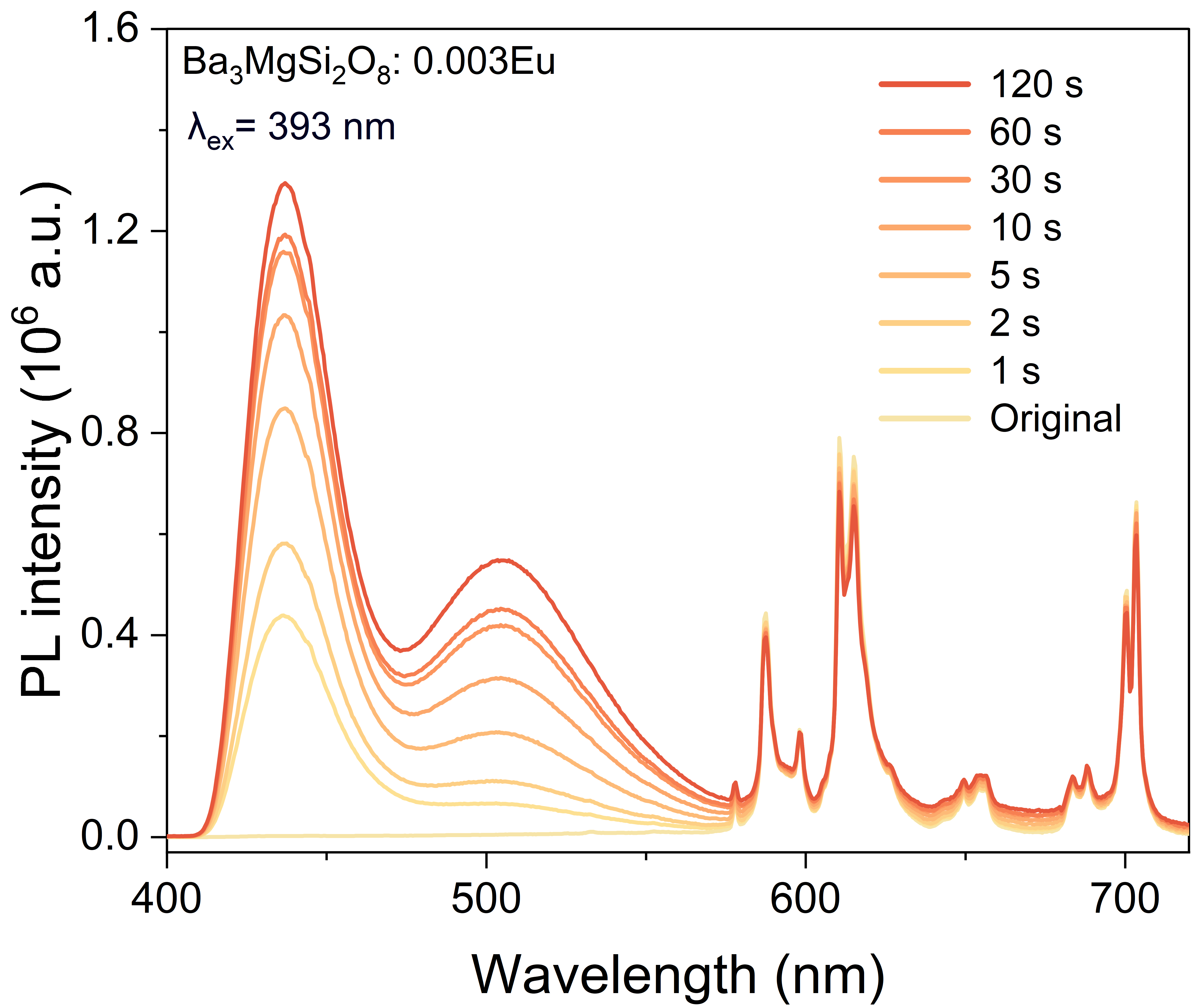


**Figure S5.** PL spectral changes (λ_ex_ = 393 nm) of BMS:0.003Eu under 265 nm irradiation with different time.


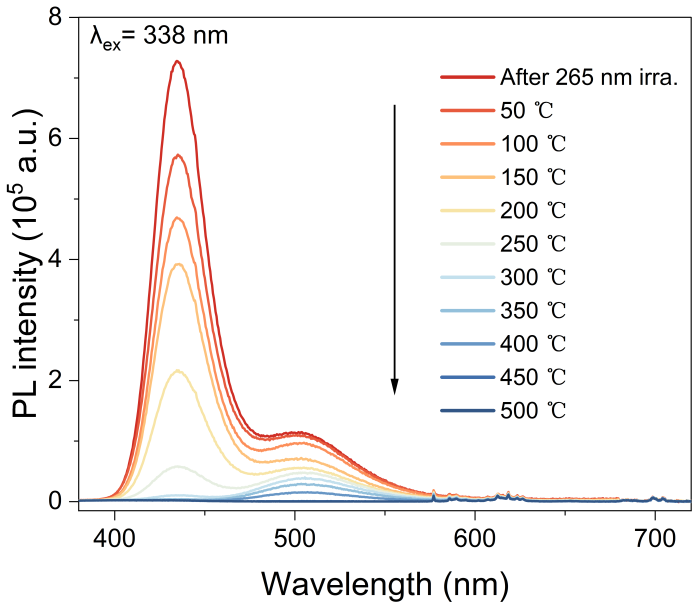


**Figure S6.** PL spectral changes (λ_ex_ = 338 nm) of the 265 nm irradiated sample (BMS:0.003Eu) with different thermal treatment temperatures


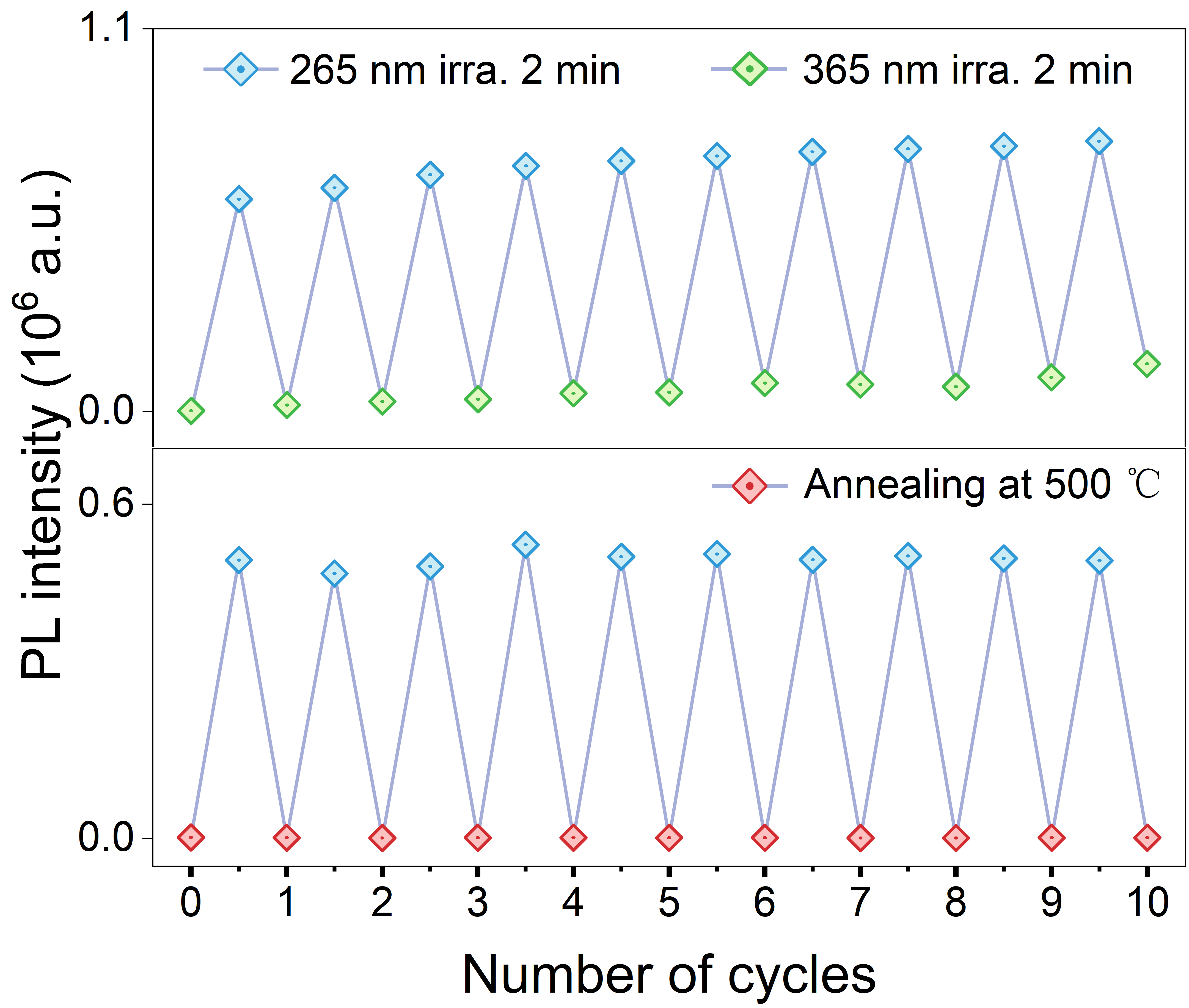


**Figure S7.** 10 reversible cycles of BMS:0.003Eu by alternating 265 nm and 365 nm irradiation (up) /thermal treatment (down).


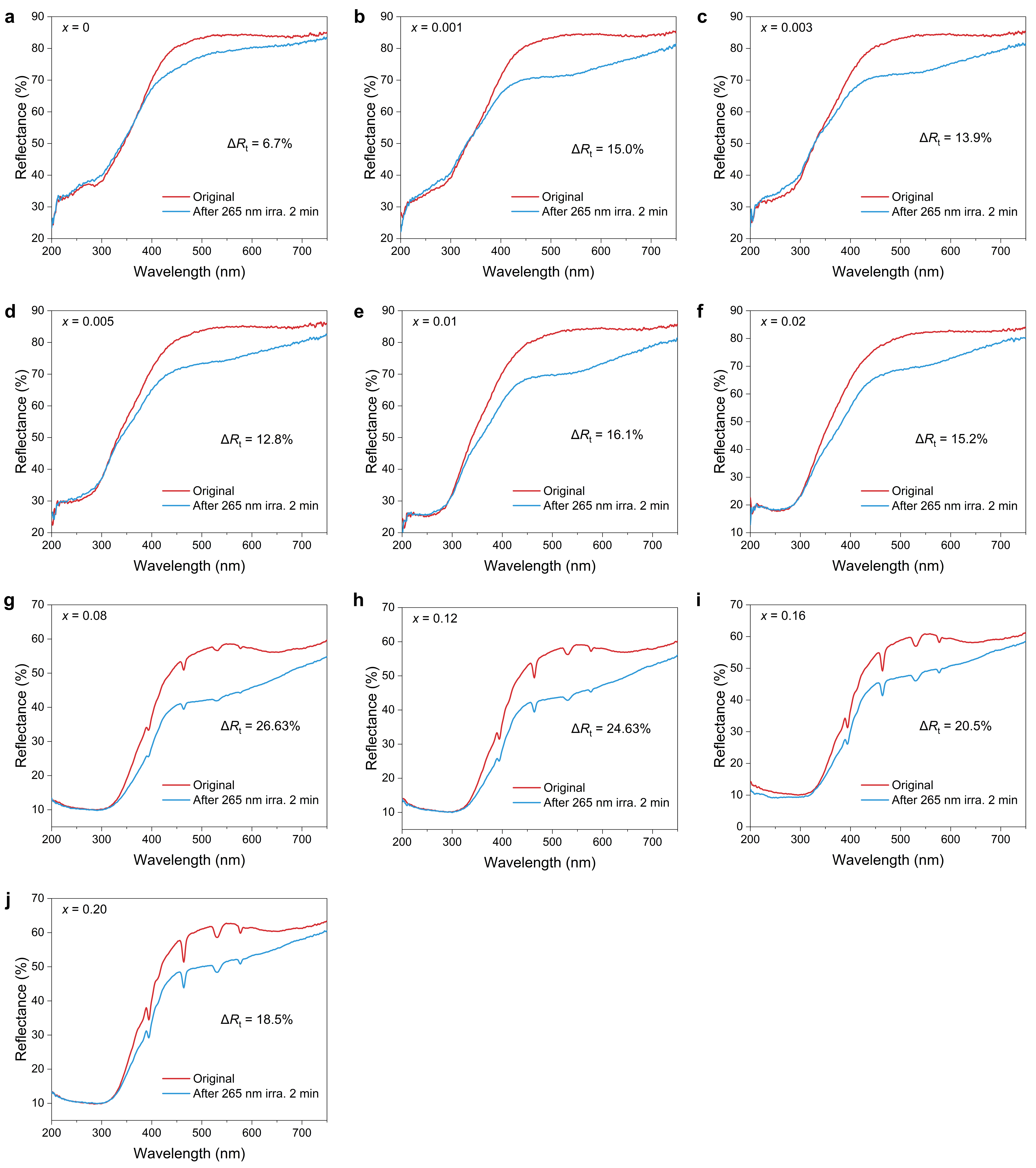


**Figure S8.** (a)-(j) Reflectance spectral changes of BMS:xEu (x = 0, 0.001, 0.003, 0.005, 0.01, 0.02, 0.08, 0.12, 0.16, and 0.20) before and after 265 nm irradiation 2 min.


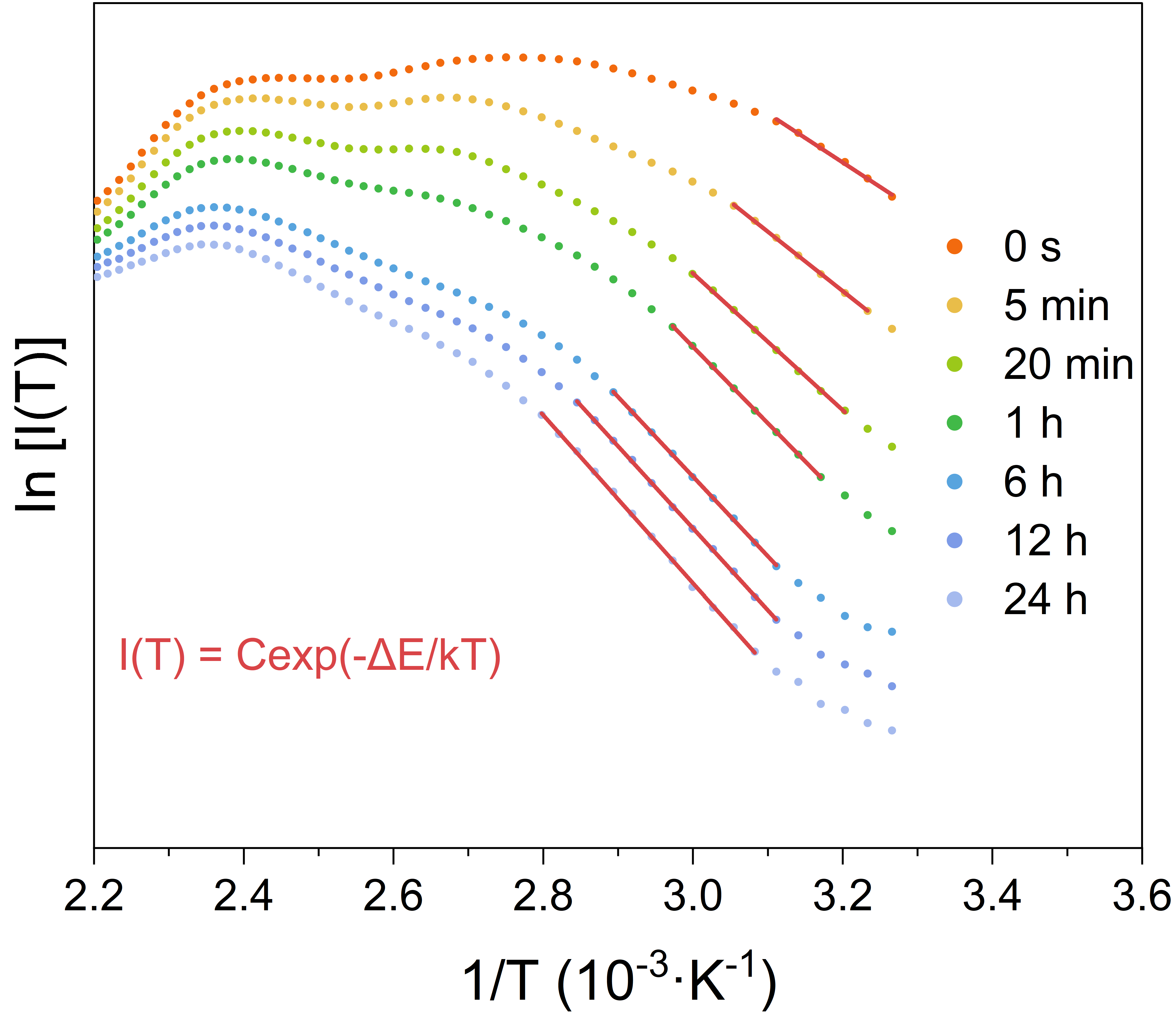


**Figure S9.** TL curves of BMS:0.003Eu analyzed by the initial rise method for evaluating the trap depths.


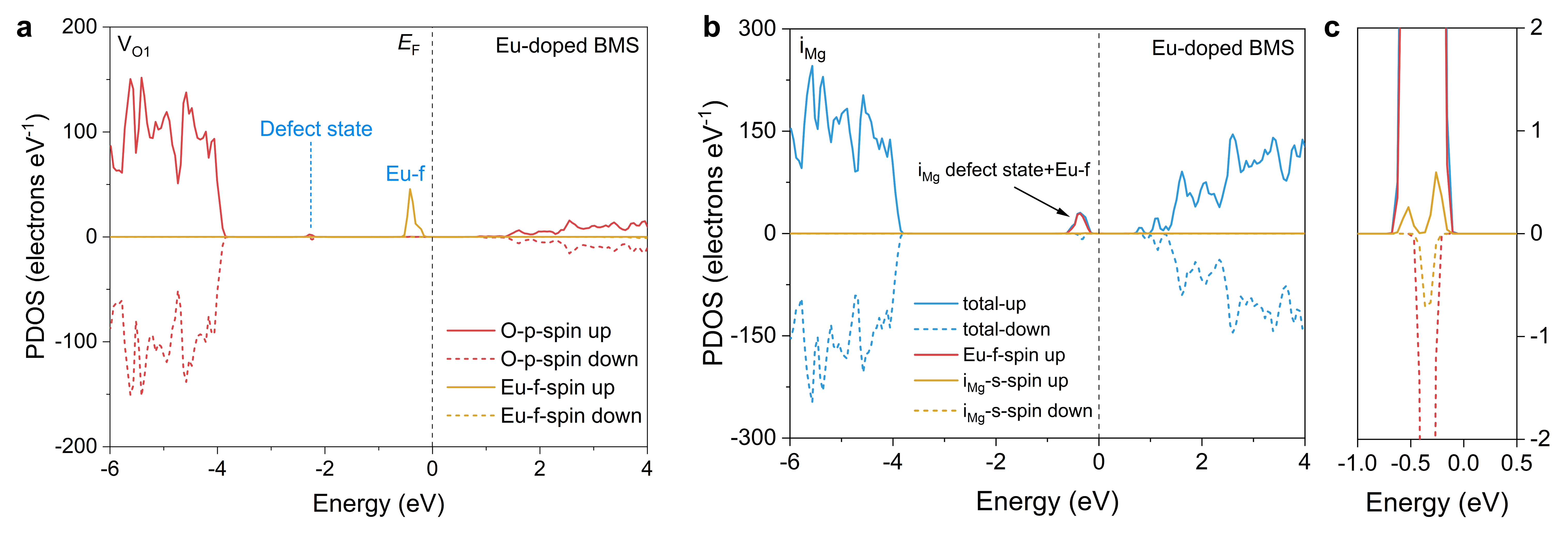


**Figure S10.** (a) The PDOS of oxygen vacancy in Eu-doped sample. (b) and (c) The PDOS of interstitial Mg in Eu-doped sample.


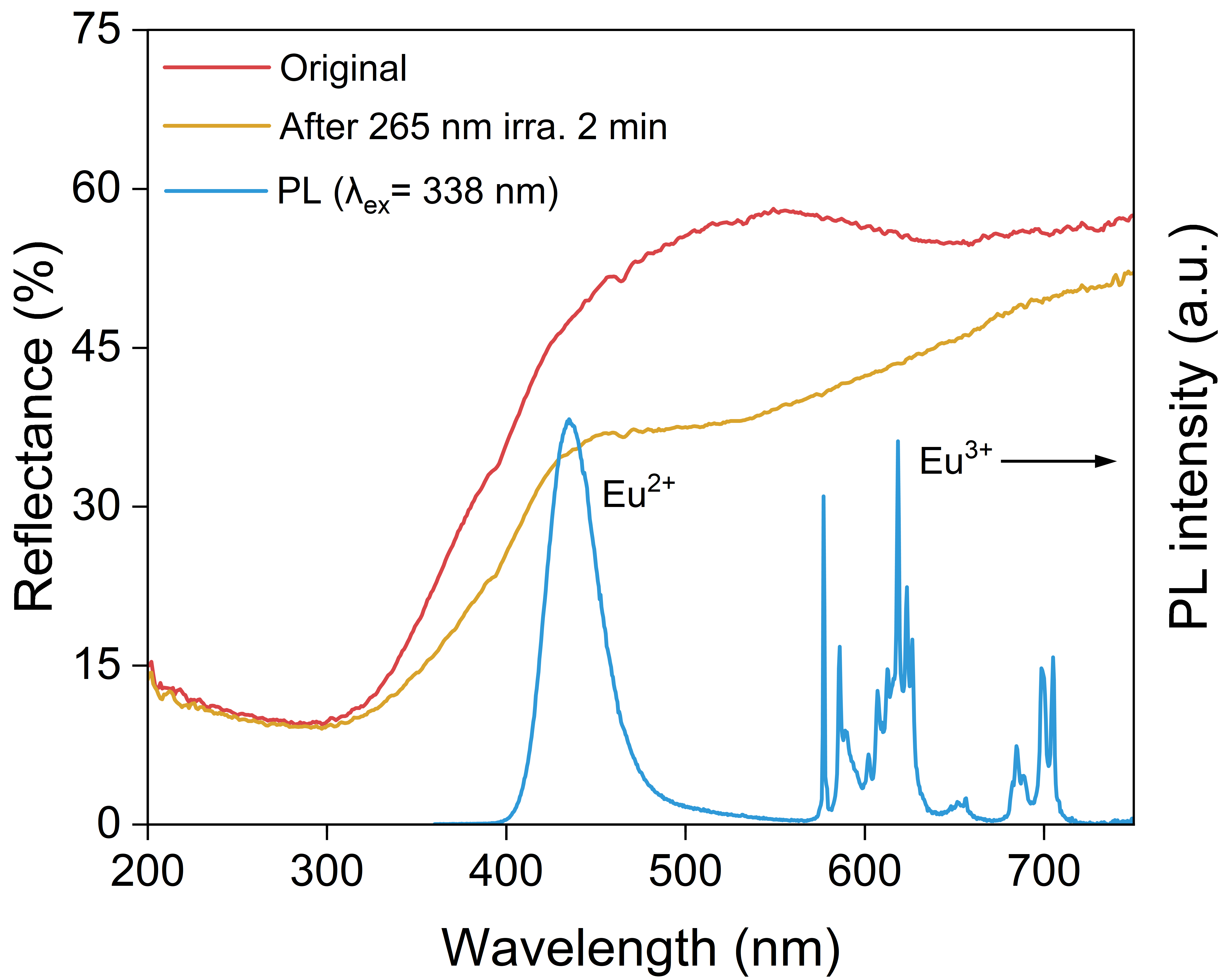


**Figure S11.** Reflectance spectra of BMS:0.04Eu before and after 265 nm irradiation for 2 min, and PL spectrum (λ_ex_=338 nm) of the irradiated sample (blue line).


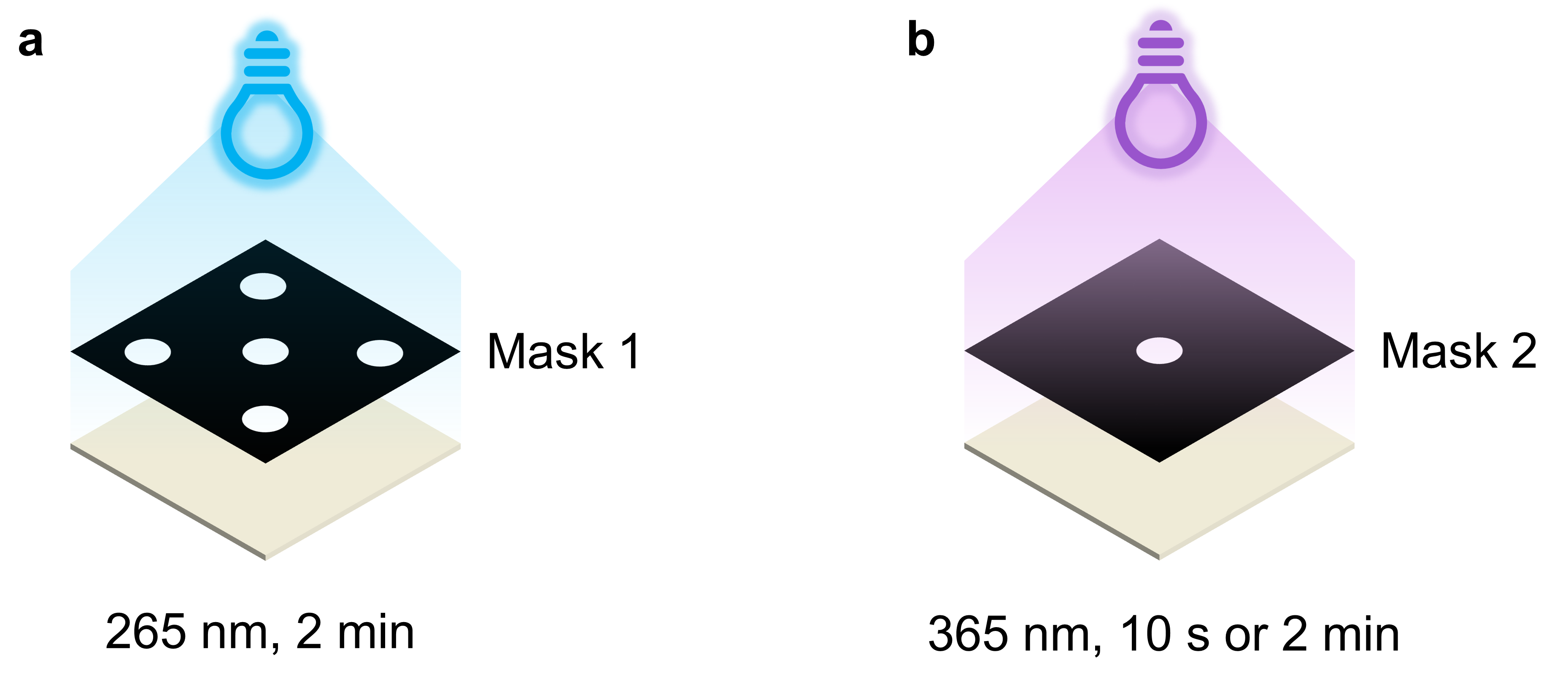


**Figure S12.** (a) The “writing” process for point matrix I. (b) The “erasing” process of output 1 or input 2.

**C. Supplementary Tables (Table S1-2)**

| **Table S1.** Mean values and standard deviations of $\Delta R_{t}$ with five experiments. | | | | | | | |
| --- | --- | --- | --- | --- | --- | --- | --- |
| **Samples** | $\boldsymbol{\Delta}\boldsymbol{R}_{\boldsymbol{t}}\boldsymbol{(\%)}$ | | | | | **Mean values** | **Standard deviation** |
|  | **1** | **2** | **3** | **4** | **5** |  |  |
| x=0 | 7.69 | 6.35 | 7.68 | 6.12 | 4.09 | 6.34 | 1.48 |
| x=0.001 | 12.04 | 10.36 | 11.54 | 11.03 | 11.25 | 11.24 | 0.62 |
| x=0.003 | 13.4 | 11.4 | 8.4 | 10.1 | 9.8 | 10.62 | 1.88 |
| x=0.005 | 12.2 | 12.46 | 12.79 | 12.86 | 11.67 | 12.40 | 0.48 |
| x=0.01 | 15.83 | 17.3 | 14.59 | 13.54 | 13.55 | 14.96 | 1.613 |
| x=0.02 | 15.4 | 16.1 | 15.08 | 13.9 | 14.77 | 15.05 | 0.81 |
| x=0.04 | 31.60 | 31.40 | 30.70 | 32.40 | 30.20 | 31.26 | 0.85 |
| x=0.08 | 30.9 | 29.95 | 23.4 | 27.7 | 25.87 | 27.564 | 3.04 |
| x=0.12 | 26.6 | 27.09 | 23.35 | 23.79 | 23.98 | 24.962 | 1.74 |
| x=0.16 | 21.4 | 20.35 | 22.6 | 21.82 | 24.54 | 22.142 | 1.57 |
| x=0.20 | 21.5 | 19.34 | 19.23 | 20.36 | 17.95 | 19.676 | 1.33 |

| **Table S2.** The defined parameters of the binary information of optical logic encryption. | | | |
| --- | --- | --- | --- |
| **Input 1**  **(365 nm 10 s)** | **Input 2**  **(365 nm 2 min)** | **Output 1**  **(Bright)** | **Output 2**  **(Dark)** |
| 0 | 0 | 0 | 0 |
| 0 | 1 | 1 | 1 |
| 1 | 0 | 0 | 1 |
| 1 | 1 | 1 | 1 |

The irradiated spot is defined as “1” and the original spot is defined as “0”. The point matrix I is defined as “0” and the point matrix II is defined as “0”.
